# Effects of chitin synthesis inhibitor treatment on *Lepeophtheirus salmonis* (Copepoda, Caligidae) larvae

**DOI:** 10.1371/journal.pone.0222520

**Published:** 2019-09-23

**Authors:** Hulda María Harðardóttir, Rune Male, Frank Nilsen, Sussie Dalvin

**Affiliations:** 1 Sea Lice Research Centre, Department of Biological Science, University of Bergen, Bergen, Norway; 2 Sea Lice Research Centre, Institute of Marine Research, Bergen, Bergen, Norway; Stazione Zoologica Anton Dohrn, ITALY

## Abstract

The salmon louse (*Lepeophtheirus salmonis*) is an ectoparasite infecting Atlantic salmon (*Salmo salar*), which causes substantial problems to the salmon aquaculture and threatens wild salmon. Chitin synthesis inhibitors (CSIs) are used to control *L*. *salmonis* in aquaculture. CSIs act by interfering with chitin formation and molting. In the present study, we investigated the action of four CSIs: diflubenzuron (DFB), hexaflumuron (HX), lufenuron (LF), and teflubenzuron (TFB) on larval molt. As the mode of action of CSIs remains unknown, we selected key enzymes in chitin metabolism and investigated if CSI treatment influenced the transcriptional level of these genes. All four CSIs interfered with the nauplius II molt to copepodids in a dose-dependent manner. The EC_50_ values were 93.2 nM for diflubenzuron, 1.2 nM for hexaflumuron, 22.4 nM for lufenuron, and 11.7 nM for teflubenzuron. Of the investigated genes, only the transcriptional level of *L*. *salmonis* chitin synthase 1 decreased significantly in hexaflumuron and diflubenzuron-treated larvae. All the tested CSIs affected the molt of nauplius II *L*. *salmonis* larvae but at different concentrations. The larvae were most sensitive to hexaflumuron and less sensitive to diflubenzuron. None of the CSIs applied had a strong impact on the transcriptional level of chitin synthesis or chitinases genes in *L*. *salmonis*. Further research is necessary to get more knowledge of the nature of the inhibition of CSI and may require methods such as studies of protein structure and enzymological studies.

## Introduction

The salmon louse (*Lepeophtheirus salmonis*) is an ectoparasitic copepod, which feeds and lives on salmonid fish. The life cycle of *L*. *salmonis* consists of eight stages [1]. Like other arthropods, *L*. *salmonis* develop and grow by ecdysis. In aquaculture, *L*. *salmonis* infection of Atlantic salmon is currently a big challenge causing high financial costs, caused by the cost of chemical treatment, reduction of salmon growth and increased risk of secondary infections [2]. Salmon farmers use several methods to control lice infestations. One of these methods is the oral administration of benzoylurea-based chitin synthesis inhibitors (CSIs). CSIs interfere with the molting process and chitin formation [3]. However, the molecular mode of action of CSIs is not clear.

**Fig 1:**
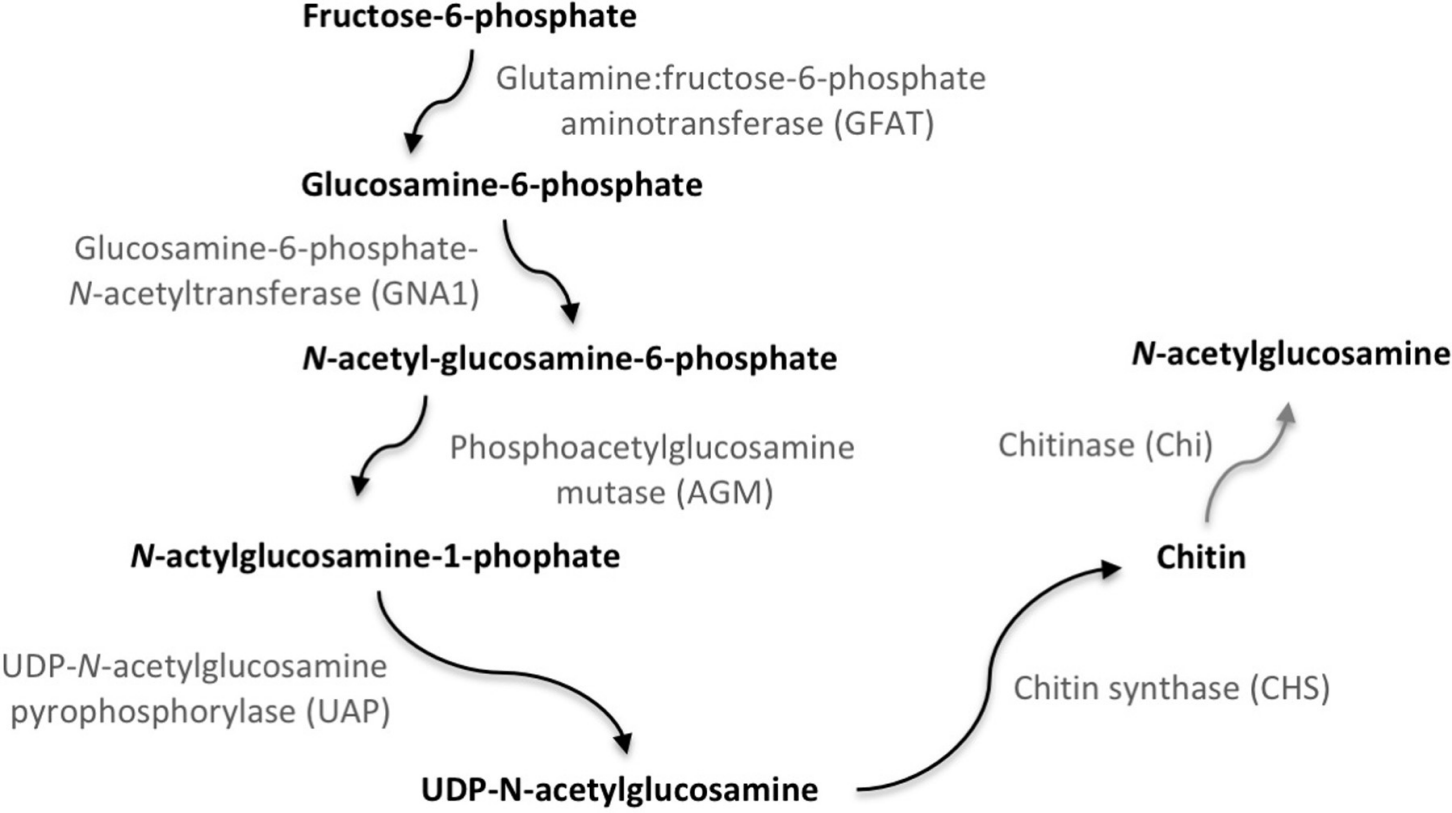
Simplified chitin pathway illustrating chitin synthesis and chitinases enzymes analyzed in the study. The chitin pathway is obtained from the amino sugar and nucleotide sugar metabolism (KEGG PATHWAY: map00520).

Chitin is a polymer of *N*-acetylglucosamine and one of the major components of the exoskeleton and peritrophic matrix of arthropods. The synthesis of a new exoskeleton takes place as the old exoskeleton is partially degraded. Chitin is a derivative of glucose and synthesized from Uridine diphosphate *N*-acetylglucosamine [4,5] (Fig 1). The reaction catalyzed by glutamine:fructose-6-phosphate aminotransferase (GFAT, EC 2.6.1.16), is one of the key steps in the chitin synthesis pathway, followed by four enzymatic reactions ending with the polymerization of chitin by chitin synthase (CHS, EC 2.4.1.16). Chitin can also be hydrolyzed into *N*-acetylglucosamine by chitinases, and *N*-acetylglucosamine can be used as a substrate for a new chitin polymer (Chi, EC 3.2.1.14).

CSIs interfere with chitin formation in the procuticle and the deposition of epicuticle, resulting in abortive molt and death [6,7]. CSIs have also been demonstrated to inhibit egg fertility and hatching [8,9]. The mode of action of CSIs has been discussed for several decades, and the enzymes in the chitin synthesis pathway have been suggested to be the target. However, little direct evidence has been reported to support this assumption. Since 1970, when the first benzoylurea was introduced on the market, many suggested that CHS could be the target of CSIs. Recently a nonsynonymous mutation in the CHS sequence has been reported in a few CSIs-resistance arthropods [10–12]. However, neither *in vivo* nor *in vitro* studies have shown inhibition of the catalytic activity of CHS by CSIs [13,14].

The CSIs, di- and teflubenzuron, are used in salmon farming in Norway [15]. Despite the many years of use, no resistance towards the drugs have been reported in *L*. *salmonis*, thus CSIs are attractive to use as other alternative chemical treatments have become less effective [16]. Other CSIs such as hexaflumuron and lufenuron have also been tested against *L*. *salmonis* [17,18].

A primary environmental concern regarding the use of CSIs is their effect on non-target arthropods in the marine environment [19]. Understanding the mode of action of CSIs in *L*. *salmonis* may offer an opportunity to develop more targeted drugs with fewer side-effects on non-targeting crustaceans. When searching for a target to study the mode of action of CSIs, the chitin pathway genes are attractive choices as CSIs interfere with chitin production and molting. The key enzymes in the chitin synthesis pathways of *L*. *salmonis* has been characterized and their expression during the synthesis of a new cuticle in preadults analyzed [20,21], and these genes were, therefore, selected to investigate the mode of action of CSIs. The object of this study was to investigate the dose-dependent relationship between the chitin synthesis inhibitors: diflubenzuron, hexaflumuron, lufenuron, and teflubenzuron and larval molting in *L*. *salmonis*, and furthermore to analyze the putative transcriptional effects on chitin synthesis and chitinases genes in *L*. *salmonis*.

## Material and methods

### Production of nauplius I larvae

The laboratory strain LsGulen of *L*. *salmonis* was maintained at low infection intensity on farmed Atlantic salmon (*Salmo salar*) [22]. The fish were hand fed on a commercial diet and reared in seawater with a salinity of 34.5 ppt with a temperature of 10°C. Eggs from gravid *L*. *salmonis* were collected from infected fish anesthetized with a mixture of methomidate (5 mg/l) and benzocaine (60 mg/l) for 3 min. Pairs of egg-strings were kept in separate wells in a flow-through system until hatching [22]. Only newly hatched nauplii I larvae (≤ 4 h old) were used for the experiment. All experimental procedures were performed following the Norwegian Animal Welfare Legislation and were approved by the Norwegian Food Safety Authority (ID8589). Salmonid fish are not expected to experience any negative effects of low level *L*. *salmonis* infections.

### Chemicals

Diflubenzuron (DFB), hexaflumuron (HX), lufenuron (LF), and teflubenzuron (TFB) of the analytical standard were supplied from Sigma-Aldrich. Stock solutions of CSIs were prepared in 100% dimethyl sulfoxide (DMSO) in glass bottles. For exposure, the stock solutions were diluted 1/1000 (0.1% DMSO) in seawater to get the required dose.

### Performance of bioassay

In 2-ml glass bottles, 20 to 30 newly hatched larvae were added together with the required concentrations of the chemicals in a total of 1.5 ml fresh seawater. The control larvae were treated with seawater containing 0.1% DMSO. The glass bottles containing the larvae were placed in an incubator (Binder) at 10°C. After 2 to 2.5 h of incubation; the larvae were collected, rinsed in seawater and placed in flow-through incubators to monitor further development [22]. The temperature in the flow-through incubators was 8.9 ± 0.4°C during the experiments.

### Pilot experiments

In the pilot experiment, the concentrations of each chemical to be used for experiments were established (see 2.5 and 2.6). Starting concentration (5 mg/l) was selected based on previous work in *L*. *salmonis* [23]. The pilot experiments were performed using dilution series (10-fold), which ranged from the starting concentrations until the lowest observed effect concentration (LOEC) for each chemical was found.

### Phenotypes

From the pilot experiment, the effective concentrations (EC) affecting ~ 95% of the treated larvae were chosen to describe the phenotype: 321.9 nM (0.1 mg/l) for DFB, 2.2 nM (0.001 mg/l) for HX, 39.1 nM (0.02 mg/l) for LF, and 26.2 nM (0.01 mg/l) for TFB. Nauplius I larvae were treated as described in (2.3), development progress was investigated one day post-treatment (dpt) and five dpt where the larvae were inspected under a microscope to confirm if nauplius I and nauplius II had molted successfully to nauplius II and copepodids, respectively. At seven dpt, a final inspection was done to include any possible delayed molt. Affected larvae were defined as individuals which did not molt or had molted to abnormal copepodids. Hereafter these individuals are referred to larvae with incomplete molting.

### Dose-response analysis and EC value

The three concentrations obtained from the pilot experiments (2.4), which gave an effect from 5% to 95% in treated-larvae were chosen to be used in the dose-response analysis. The dose-response analysis was analyzed at seven dpt by counting normal larvae and larvae with incomplete molting at the end of the bioassay (see 2.3). Three independent experiments, each with four biological replicates containing 20–30 larvae each were performed. The EC_50_ values were defined as the concentration where incomplete molting was observed in half of the treated animals. The average percentage of incomplete molting obtained from each of the four biological replicates were plotted against concentration of the chemical. Linear regression was performed for each of the three experiments to produce dose-response curve and EC_50_ values were calculated from the average values obtained from the trendlines.

### Collection of different instar ages of *L*. *salmonis* larvae for transcriptional analysis

The transcriptional levels of chitin synthase 1 (LsCHS1, Genbank accession number: MH350851), chitin synthase 2 (LsCHS2, MH350852), glutamine:fructose-6-phosphate aminotransferase (LsGFAT, CDW19749.1), glucosamine-6-phosphate *N*-acetyltransferase (LsGNA1, MH370500), phosphoacetylglucosamine mutase (LsAGM, MH320769), UDP-*N*-acetylglucosamine pyrophosphorylase (LsUAP, ACO12073.1) and chitinases (LsChi1 (KM668222.1), LsChi2 (AIE4595.1), LsChi4 (KJ361515.1)) in *L*. *salmonis* were analyzed in nauplius I and II, and in nauplius II treated with CSIs. The development of *L*. *salmonis* is highly dependent on water temperature [24]. At 9°C the following instar-ages of nauplius I was collected: early (2 h post-hatching), middle (13 h post-hatching), and late (24 h post-hatching). For nauplius II: early (27 h post-hatching), middle (72 h post-hatching), and late (110 h post-hatching). For expression analysis during the instar-ages, each biological replicate contained larvae from a single egg-string (approximately 300 larvae). For CSI experiments, newly molted nauplii I larvae were treated as described in 2.3. Treated and control (DMSO only) groups originated from the same egg-string and for each sample contained approximately 100 larvae. The investigations were performed at 8.9 ±0.4°C and sampling time was adjusted accordingly to degree hours or days. All experiments contained five biological replicates. After harvest, the samples were stored in RNAlater (Qiagen) for at least one day at 4°C before RNA extraction or for long time storage at—20°C.

### RNA extraction and cDNA synthesis

RNA isolation was carried out according to the Tri Reagent® Protocol (Sigma-Aldrich) with some modifications: homogenization of the samples was performed in Tri-reagent (Sigma-Aldrich) using 1.4 mm zirconium oxide beads (Precellys 24) and a TissueLyser LT (Qiagen) for 7 min at 50 Hz. The RNA was extracted as described before [24]. The purified RNA was dissolved in RNase free water and quantified using a Nanodrop 2000 Spectrophotometer (NanoDrop Products). RNA samples were either frozen at—80°C or used directly for the production of complementary DNA (cDNA). For cDNA synthesis, the samples were DNase treated according to the manufacturer´s protocol (Turbo DNase free^TM^ kit, Ambion Foster City, CA). RNA (200 ng) was reverse transcribed to cDNA using the Affinity Script cDNA Kit (Agilent) according to the manufacturer’s instructions. Before storage at—20°C the cDNA was diluted ten times for quantification of transcript levels described in (2.9).

### Quantitative RT-PCR (qPCR)

The transcriptional levels of selected genes were quantified by qPCR using PowerUp^TM^ SYBR Green® Fast Universal PCR Master Mix; Applied Biosystems. The primers and their efficiency were adapted from a previous study [20], and are listed here in Table 1. The salmon louse elongation factor 1α (eEF1α) was used as a reference gene [25]. Reaction specificity was verified by the presence of a single peak in the melting curve. For each experiment, five biological replicates were analyzed each with two technical replicates. One sample without reverse transcriptase enzymes was included to exclude contamination with genomic DNA. Thermal cycling and quantification were done on the Applied Biosystems 7500 Fast Real-Time PCR System in 10 μl reactions under standard conditions (initiation: 50°C for 2 min, holding at 95°C for 2 min, 40 cycles at 95°C for 15 seconds then 60°C for 1 min). The relative quantification analysis was performed by calculating the difference in threshold cycle (Ct) between the gene of interest and the eEF1α, and relative expression was calculated using the control of each experiment as standard. 2^-ΔΔCt^–method was used to analyze the fold difference of gene expression between the CSIs-treated samples and control samples [26].

**Table 1:** List of qPCR primers. Products size and efficiency are listed for each primer product (SYBR Green assay).

Gene	Forward primer (5´-3´)	Reverse primer (5´-3´)	Size (nt)	Efficiency
LsAGM	GGATGGAGATCGAATTGCTACATTG	TGACCATTTGCCTCAAAGTAAACTC	234	85–87%
LsChi1	TCTCAAGTCTGTCTCTATTCCTCCT	TTTACCATTGCTCCAACGATAAGTG	187	94–100%
LsChi2	GTCACAATGCTCCTCTCTATGCTCC	TCCTGTTTTGCCTCCATCCTTTGAA	209	86–100%
LsChi4	TAACCCACCCTGTCTACGCA	ACGAGGATGTGGACGGATTC	74	90–99%
LsCHS1	GCGTTGCGTTCATACCTTCT	TAATTTTCCCACCAACCCGC	214	99–100%
LsCHS2	TCACTCACGTCCCCATTTCT	TCGATGGATGCTAGCCGAAT	242	83–90%
LsEL1α	CATCGCCTGCAAGTTTAACCAAAT	CCGGCATCACCAGACTTGA	155	93–95%
LsGFAT	AATAGTTGCTGCTCGTCGTG	TCAGAGGCAGAGTCCATTCG	210	88–99%
LsGNA1	CATTACAATCAGGAGATGATGAGCG	CAATGACAATGACGTAGTAGGTTCC	144	96–98%
LsUAP	GGAGGATTATACGAGGCGCT	ATAAGCACCGTCCACTTTGC	228	88–99%

### Data analysis and statistics

Statistical analysis was performed using the SPSS statistics software V.21 (IBM#SPSS# Statistics, Armonk, NY, USA). A significance level of P < 0.05 was used for all tests. Values from the expression analysis during the instar ages of *L*. *salmonis* larvae were subjected to one-way analysis of variance (ANOVA). For the CSI experiments, paired Student´s t-test was used to analyze the difference in the Ct values between treated and controls larvae.

## Results

### Phenotypic analysis of CSI-treated larvae

**Fig 2:**
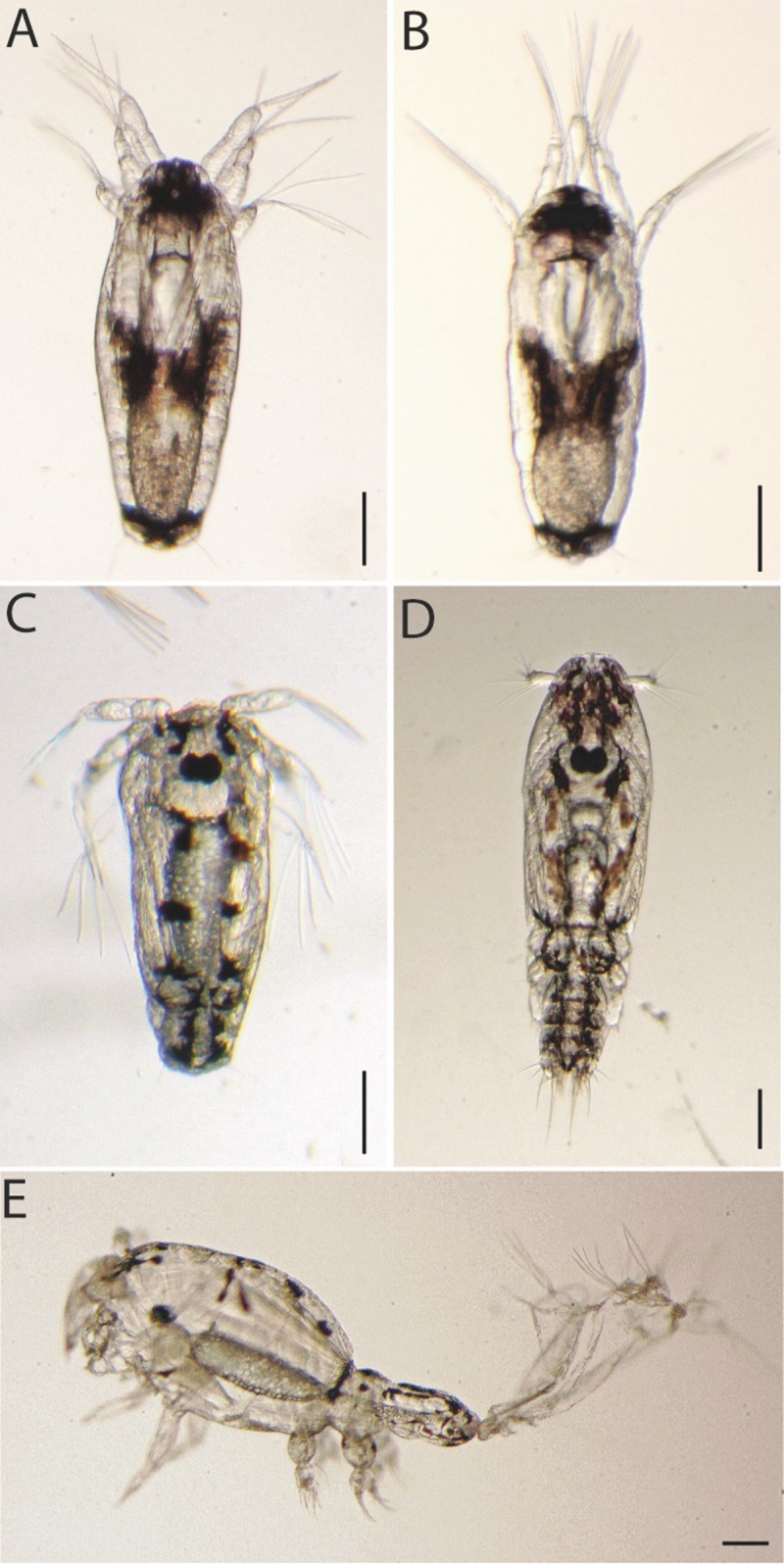
*Lepeophtheirus salmonis* larva treated with chitin synthesis inhibitors. Nauplii I larvae were treated with EC_~95_ of CSIs for 2 h. Only the lufenuron-treated larvae are shown as the four CSIs resulted in identical phenotype. Lufenuron-treated nauplius II (A) and control nauplius II (B), one day post-treatment (dpt). Lufenuron-treated molt arrested larvae (C) and control copepodid (D), five dpt. Lufenuron-treated copepodid, seven dpt (E). Control copepodid for at seven dpt looks identical to copepodid at five dpt (D). Scalebar indicates 0.1 mm.

From the pilot experiment, doses affecting ~ 95% of the treated larvae were used to analyze the phenotypes of CSI-treated larvae (see 2.4). All CSIs induced similar effects on larvae molting and appearance. At one dpt, all treated-nauplii I larvae had molted to nauplii II; however, the position of the antenna in newly molted individuals was altered (Fig 2A) compared to controls (Fig 2B), and the antenna vibrated continuously in the treated larvae. From two dpt, treated nauplii II exhibited little or no activity compared to control nauplii II (S1 File); but had normal morphology of antenna. At five dpt, all control larvae had molted to copepodids (Fig 2D), whereas less than 5% of the CSI-treated larvae had completed normal molt from nauplii II to copepodids (Fig 2C). At seven dpt, a few CSIs treated-larvae had molted but exhibited gross abnormal morphology (Fig 2E). Visual inspection of live animals revealed that the exoskeleton of treated copepodids were more transparent and flexible than control copepodids. The copepodids were bloated, unable to swim, vulnerable to handling, and died between eight to ten dpt.

### Determination of EC_50_ values for molt to copepodids

**Fig 3:**
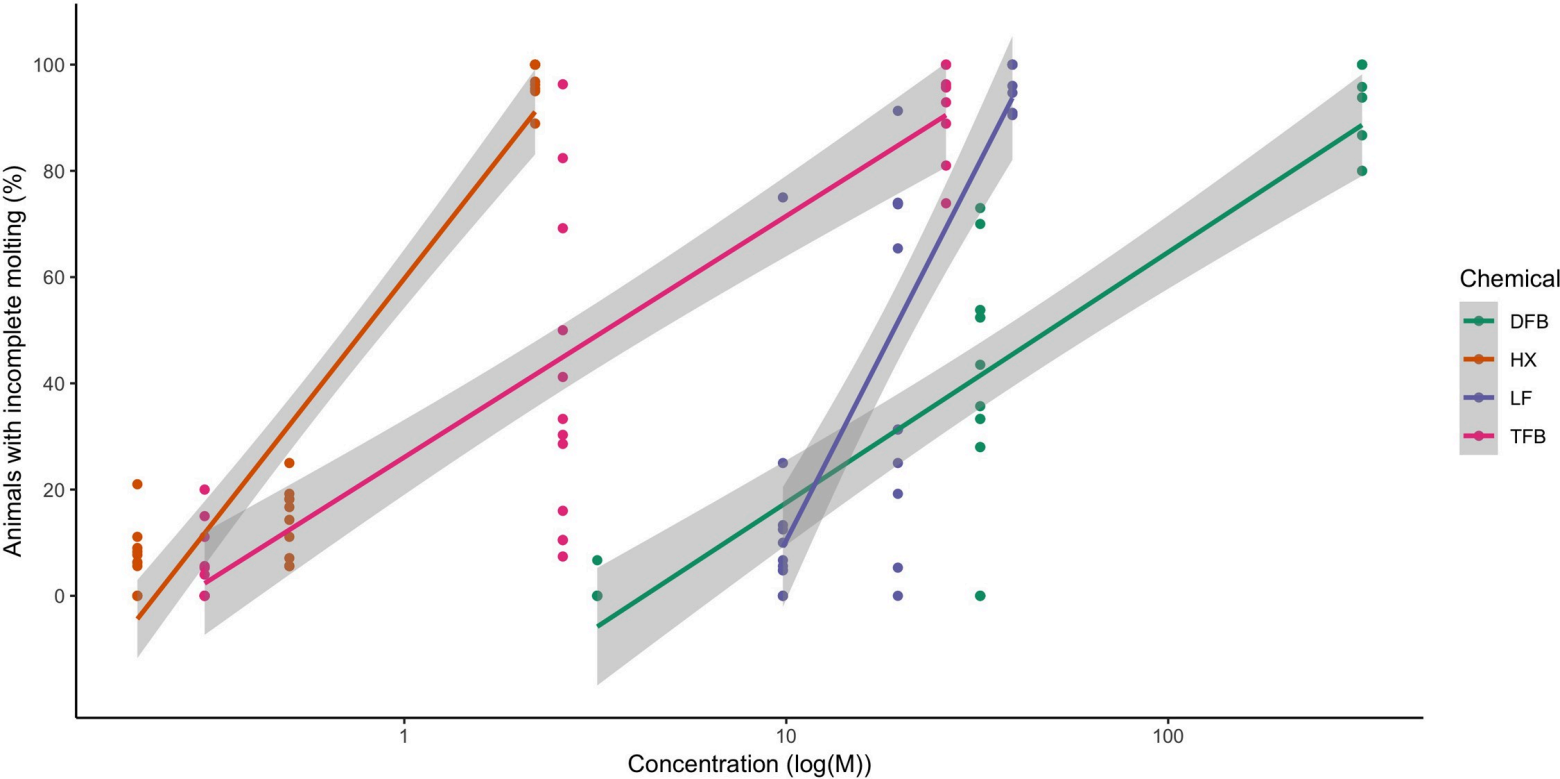
Molting success in *Lepeophtheirus salmonis* after treatment with chitin synthesis inhibitors. Dose-response analysis of three different concentrations of diflubenzuron (DFB), hexaflumuron (HX), lufenuron (LF) and teflubenzuron (TBF). The graph shows the dose-response relationship between the chitin synthesis inhibitors and the inability to perform molt from nauplius II to copepodid, five days after treatment. The results are from all replicates (four biological and three technical) for each chemical. The concentrations (nM) are in log scale.

As the molt from nauplius I to nauplius II was not affected by the CSIs the dose-response relationship of the four CSIs was determined as the ability to molt from nauplius II to copepdid. Based on the pilot experiments, three doses were tested for each chemical (Table 2). Newly hatched nauplii I larvae were treated with the CSIs, and the effects on molting to copepodids were observed at seven dpt, two days after all the control larvae had molted to copepodids. The CSIs did not affect the developmental speed of nauplius I. DFB, HX, LF, and TFB all inhibited the molt from nauplius II to copepodid in a dose-dependent manner (Fig 3). The larvae were most sensitive to HX and least sensitive to DFB. The EC_50_ values were calculated from the linear regression obtained from the dose-response curves (Table 2).

**Table 2:** List of chitin synthesis inhibitors. The concentrations used in the dose-response analysis and the EC_50_ with SD values are showed.

		Concentration (μg/l)		EC_50_ (nM)	SD (+/-)
Hexaflumuron	1.00	0.25	0.10	1.20	0.02
Diflubenzuron	100.00	10.00	1.00	93.17	49.14
Lufenuron	20.00	10.00	5.00	22.36	2.92
Teflubenzuron	10.00	1.00	0.10	11.73	0.99

### The relative expression of chitin synthesis and chitinases genes during the molt cycle

**Fig 4:**
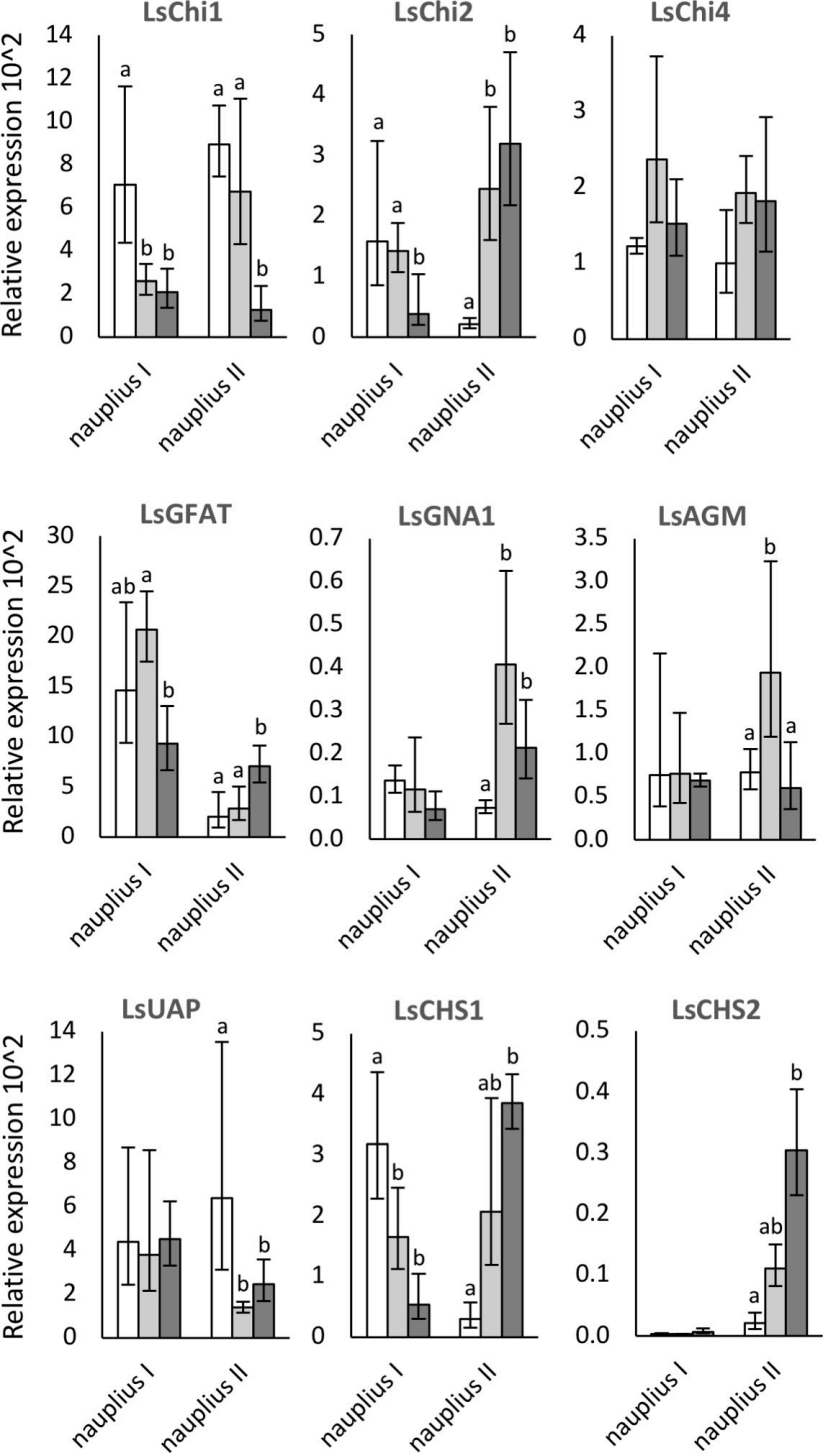
Relative expression of chitin synthesis and chitinases genes in *Lepeophtheirus salmonis* nauplius. Transcriptional levels of LsChi1, LsChi2, LsChi4, LsGFAT, LsGNA1, LsAGM, LsUAP, LsCHS1, and LsCHS2 were analyzed in early (white), middle (grey), and late (dark grey) nauplius I and II instars. Values are expressed as mean ± confidence intervals of five biological replicates. Statistical analysis was performed using one-way ANOVA. Letters indicate statistically significant difference between samples.

Expressional levels of chitin synthesis and chitinases genes were analyzed in early, middle, and late instar-ages of nauplius I and II (Fig 4). The expression patterns during the molt cycle differed between nauplius I and II for all analyzed genes, except for LsChi4 where no significant regulation was detected. The expression pattern of LsChi1 is similar in nauplius I and nauplius II with decreasing expression towards molt. The expression pattern of LsChi2 exhibits an opposite trend in the two nauplii stages with decreasing expression towards molt in nauplius I and increasing in nauplius II.

The transcriptional level of LsGFAT is somewhat similar to the LsChi2 expression pattern. The transcriptional level of LsGNA1, LsAGM, and LsUAP were unchanged in the three selected instar-ages of nauplius I. However, in nauplius II the transcriptional level of LsGNA1 and LsAGM increased from early to middle instar, while the LsUAP transcripts decreased. Similar to LsChi2 the transcriptional level of LsCHS1 decreased from early to middle instar in nauplius I, followed by an increased from early to late instar in nauplius II. LsCHS2 transcripts were almost undetectable in nauplius I whereas the level of transcription increased from early to late nauplius II instar.

### Effect of CSIs on the expression of chitin synthesis and chitinase genes in *L*. *salmonis* larvae

**Fig 5:**
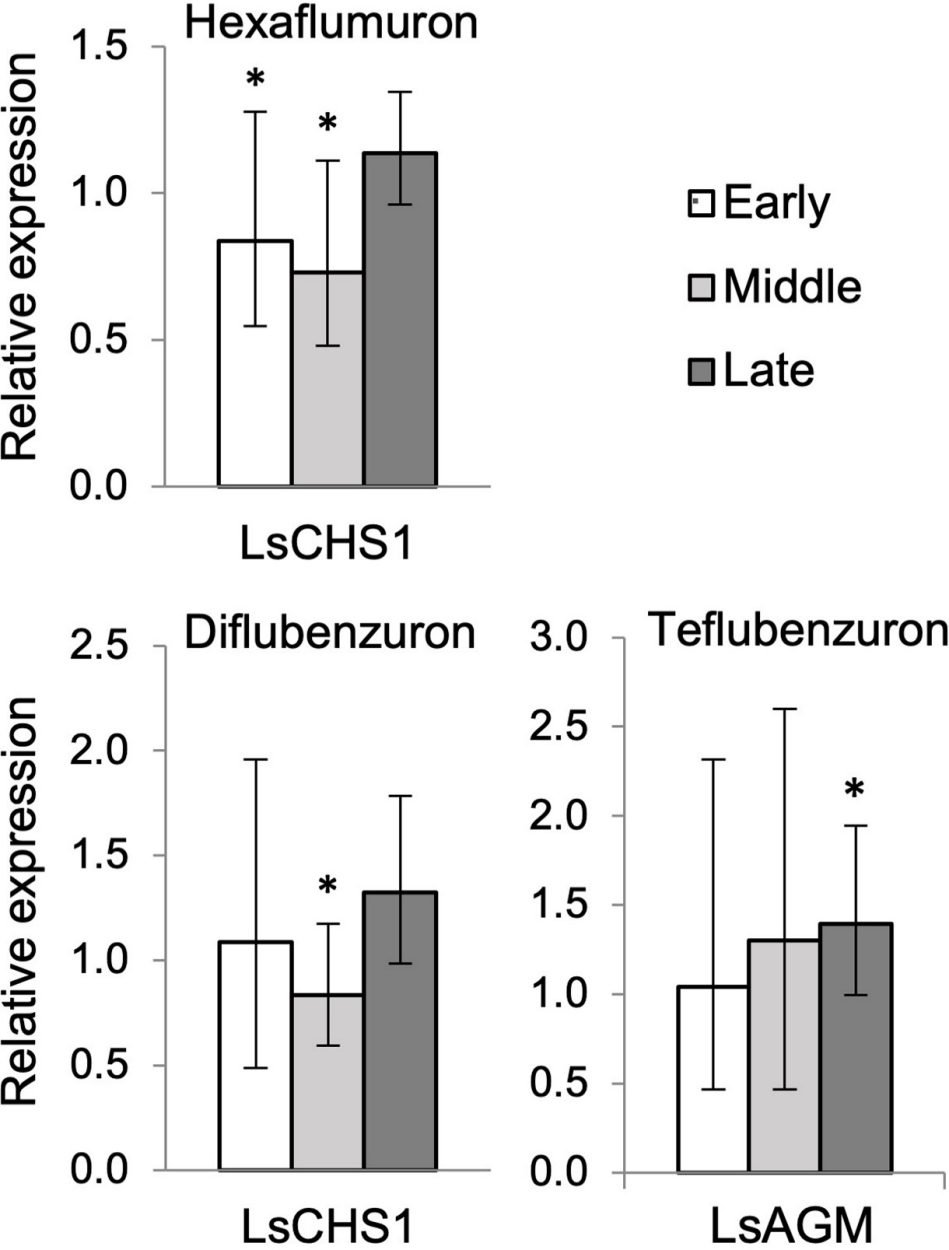
Transcriptional changes induced by chitin synthesis inhibitors. Transcriptional levels were analyzed in early, middle, and late instar of nauplius II treated with chitin synthesis inhibitors (CSIs). Only genes and treatment combination with significantly different expression in CSI-treated nauplius II are shown. Transcription in untreated nauplius II was set to 1. Values are expressed as mean ± confidence intervals of five biological replicates. Statistical t-test was used to evaluate the significant differences indicated by (*) in the transcriptional level between treated and control larvae.

To investigate if CSIs interfere with the expression of chitin synthesis or chitinases genes in nauplii II larvae, newly hatched nauplii I larvae were treated with EC_~95_ of the four inhibitors. Overall all four CSIs had a negligible impact on the transcriptional level in all three investigated instar-ages of nauplius II. Of the nine genes tested only LsCHS1 and LsAGM exhibited significantly different expression with low fold (< 2-fold) changes (Fig 5. only differentially expressed genes are shown, S2 File). The transcriptional level of CHS1 was significantly lower in the middle instar of DFB-treated nauplius II, and both in early and middle instar of HX-treated nauplius II. Aslo, the transcriptional level of LsAGM was significantly higher in the late instar of TFB-treated nauplius II. No significant changes were obtained in the transcriptional level of LF-treated nauplius II.

## Discussion

Many studies have shown that CSIs interfere with chitin synthesis and that younger stages of arthropods are more sensitive to CSIs than older stages [3,27,28]. Also, for *L*. *salmonis* the immature stages are more vulnerable to CSIs treatment than adult lice [29,30]. Despite many years of studies of the mode of action of CSIs, specific inhibition of a target enzyme has not yet been demonstrated.

In this study, most the *L*. *salmonis* nauplius II affected by the CSIs were unable to shed the old exoskeleton and the few larvae that did complete the molt to copepodids appeared to have more flexible and transparent exoskeleton than the control larvae. These results are similar to observations of molt in immature stages of other arthropods, e.g., the estuarine mud crab (*Rhithropanopeus harrisii*) [31], the potato beetle (*Leptinotarsa decemlineata*) [32], and the calanoid copepod (*Eurytemora affinis*) [33]. CSIs have also been shown to affect the structure and composition of the exoskeleton [34–37]. A recent study in *L*. *salmonis* showed that lufenuron-treated nauplius had a less electron-dense layer, poorly organized epicuticle, and a diffuse procuticle [17], which could be a result of less chitin production and could explain the flexible cuticle structure of the affected copepodids observed in the present study.

As CSIs interfere with molt, the effects of CSIs on the larvae were investigated during the molting process to obtain dose-dependent relationship using non-toxic doses [38]. Similar to other studies [32,39,40], the EC differed between the four tested chemicals. Also, as observed in both insects and crustaceans, TFB was found to be toxic in lower doses than DFB [39,41,42]. EC on the ability to molt, sometimes in older studies defined as lethal concentration (LC) as an inability to molt which cause the death, have been estimated at different development stages of arthropods [8,43,44]. In *L*. *salmonis*, an EC_50_ of acute toxicity has been estimated for the preadult stage [23]. This EC_50_ value was 500-fold higher than the EC_50_ value for molt obtained in the present study. This difference is likely to be caused by the different evaluation where animals undergoing molt is expected to be more sensitive to this group of chemicals. In addition, because of the stage difference. Of the four investigated CSIs, HX (EC_50_ = 1.2 nM) was the most effective against larval molting to copepodids and around 78 times more effective than DFB (EC_50_ = 93.2 nM) the least effective CSIs tested. The same order of toxicity is reported in insects: *L*. *decemlineata* [32] and the raisin moth *(Ephestia figulilella)* [40].

Due to environmental concerns testing of CSIs has been performed in several crustaceans, including Cladocera, Copepoda and Malacostraca [33,38,42–44]. Crustaceans have also been shown to followed dose-dependent mortality during and after the molt [31,33,45]. Most of the CSIs-experiments with DFB used long term exposure (24–96 h) [38]. Compared to the harpacticoid copepod (*Tisbe battagliai)*, *L*. *salmonis* was sensitive to lower doses of TFB [42]. The EC_50_ value of DFB obtained in the present study is similar to the EC_50_ value obtained for 1^st^ instar *Daphnia magna* [44]; however, the exposure time there was 48 h, which could indicate that *L*. *salmonis* larvae are more sensitive to DFB than *D*. *magna* [46]. Molt to the first copepodid stage of the calanoid copepod (*Eurytemora affinis*) exposed to DFB a five times lower EC_50_ (LC_50_) value than in the present study most likely caused by the long exposure time (48 h) [33]. The survival of larvae of *R*. *harrisii* and the marsh crab (*Sesarma reticulatum*) decreased at 0.001 mg/l, and 0.003 mg/l, respectively, which is similar to the LOEC of DFB observed in this study [31]. Overall the ECs appear to be similar in many arthropods, and the main cause of the difference in results is likely to result from different combinations of exposure times and concentrations.

Low exposure dose inhibiting the molt of larvae was also used to see if CSIs affect the transcriptional level of chitin synthesis and chitinases genes in *L*. *salmonis*. The expression pattern of chitin synthesis and chitinases enzymes in nauplius I and II were analyzed as molt related enzymes are likely to be regulated during the molt cycle. In this study, the expression pattern of LsChi1 in nauplii larval stages was different from the pattern reported preadult I stage [20]. For LsChi2, however, the expression patterns observed in nauplii II and preadult I lice were similar but different from nauplius I expression pattern. The differences in the expression pattern of LsChi1 between nauplii stages and preadult I stage may suggest multiple functions of LsChi1 during the development. The functional role of LsChi2 have been described, and it has been suggested that LsChi2 could play a role in the remodeling of the exoskeleton in larvae [21]. Further studies unraveling the possible differentiation of two chitinases and their role in molt are warranted. In other crustaceans, chitinases are highly up-regulated during the premolt stages (D0-1), e.g., *Eriocheir sinesis* chitinase- 2, 4, and 6, *Litopenaeus vannamei* Chi2 and *Penaeus monodon* chitinase- 3 and 4 [47–49]. Expression of these chitinases are similar to the expression observed for LsChi2. The LsChi4 expression was not regulated according to molt, and its functional role could be molting independent, such as having a role in immunity as observed for one of the chitinases in *L*. *vannemei* [50].

Few studies have investigated the expression pattern of GFAT, GNA1, AGM, and UAP in crustaceans. Only in crayfish (*Cherax quadricarinatus*) has the expression of these genes been reported during the molt cycle [51]. In this study, the expression patterns of LsGFAT differed between nauplii I and II stages. Also, the expression pattern in preadult I lice was different from both nauplius I and II [20]. For LsGNA1, the expression pattern changed during the molt cycle in nauplius II, while in nauplius I and preadult I the expression patterns did not change. The expression pattern of LsAGM in nauplius I and in preadult I male did not change during the molt cycle, while in nauplius II and preadult I female the expression pattern changed during the molting cycle. The expression pattern of LsUAP showed a similar expression pattern to LsAGM in nauplius I and preadult I male; however, the expression patterns between nauplius II and preadult I female were different from each other. In *C*. *quadricarinatus*, the highest expression level of GFAT transcripts was found in postmolt- and intermolt stages, similar to the expression pattern of nauplius I. Both in *C*. *quadricarinatus* and *L*. *salmonis* nauplius II the expression levels of UAP transcripts were higher in postmolt than in premolt animals. In nauplius I, the expression patterns of LsGNA1, LsAGM, and LsUAP were similar to the expression found in *C*. *quadricarinatus*, and these expression patterns could not be related to molting events. In nauplius II, the expression patterns of these transcripts changed during the molting cycle; however, the expression levels were highest in middle of the molt cycle, which could be assumed to be the intermolt stage, and in the intermolt stage no molting events occur. In insects, GNA1 and AGM were described as “housekeeping” genes as the expression of these genes did not change during the molt cycle. Overall there is no clear trends in the expression pattern of LsGFAT, LsGNA1, LsAGM, and LsUAP during the molt cycle in *L*. *salmonis*, which indicates that these genes could be regulated independently from molting events.

The transcriptional level of LsCHS1 and LsCHS2 showed molt coordinated expression patterns with increasing expression from postmolt to premolt when the synthesis of chitin into the cuticular layers occurs. Similar expression of LsCHS1 was found in a previous study of preadult I lice where the highest expression of LsCHS1 was found in premolt lice [20]. However, in nauplius I the transcriptional level of neither LsCHS1 nor LsCHS2 was related to molting. In the crustaceans, *L*. *vannamei and C*. *quadricarinatus*, CHS transcripts increased during the premolt (D2) stage. Up-regulation of CHS1 during the premolt stages in response to ecdysone pulse is reported in insects [52]. Ecdysone is present in *L*. *salmonis* but changes in levels during instar ages has not been investigated [53].

All the tested CSIs interfered only with the *L*. *salmonis* molt from nauplius II to the copepodid, not nauplius I to nauplius II. A similar result was also observed in *T*. *battagliai*, which different from *L*. *salmonis* goes through several nauplii stages [42]. These results indicate that molt between nauplii stages may be differently regulated than the molt from nauplius to copepodid. This suggestion is also supported by our findings that LsCHS1, LsCHS2, and the three *L*. *salmonis* chitinases were not correlated with molt in nauplius I, while these transcripts were molting regulated in the nauplius II molt cycle.

CSIs had a negligible impact on the transcriptional level of chitin synthesis and chitinase genes in nauplius II. No significant difference was found in the transcriptional levels in LF-treated nauplius II, while for DFB, HX, and TFB only slightly up- or down-regulation were found in one investigated gene. These results are in accordance with other studies where the transcriptional level of chitin metabolism genes was not affected by CSI treatment [17,54]. In this study, LsCHS1 was the only gene that showed a different transcriptional level in more than one CSIs tested. The level of LsCHS1 transcripts was slightly down-regulated in both HX- and DFB-treated nauplii II larvae. Earlier studies of the effect of CSIs on CHS similarly do not show distinct patterns. In insects, the level of CHS1 transcripts in both the mosquitoes (*Anopheles quadrimaculatus*) and the mite (*Panonychus citri*) increased after DFB treatment [55,56], while no changes were observed in the red flour beetle (*Tribolium casaneum*) and the fruit fly (*Drosophila melanogaster*) [7,54]. Also in crustacea, the level of CHS1 transcripts in the TFB-treated lobster (*Homarus gammarus*) was down-regulated when exposure to a low dose of TFB, while significantly up-regulated (4.7-fold) when exposed to a high dose [19]. Many studies have shown that CSIs reduce the amount of chitin in the cuticle [36,37], and also show an accumulation of *N*-acetylglucosamine, the precursor of chitin [3]. From these results, it could be suggested that the primary target of CSIs is CHS the last enzymes in the chitin synthesis pathway although it may not affect its transcription.

Recent studies reported the same nonsynonymous mutation in the CHS gene of the CSI-resistant arthropods: the mite (*Tetranychus urticae*), the moth (*Plutella xylostella)*, and the thrip (*Frankliniella occidentalis*) [10–12]. This mutation is a substitution changing isoleucine to methionine or phenylalanine, and is not found in the catalytic active site of CHS but in a C-terminal transmembrane region, which is highly conserved, and could play an essential function for the enzyme. Further studies were performed in *D*. *melanogaster* using a genome-editing approach where the nonsynonymous mutation in the CHS gene (named *kkv*) was confirmed to introduced CSI resistance [11]. These findings show that CHS is likely the target of CSIs and the position of the nonsynonymous mutation in the CHS sequence could be the reason why an inhibition towards the catalytic activity of CHS has not been found previously [10].

In conclusion, further investigation of the mechanism of CSIs on CHS and their effect on its function: possibly involving localization of the enzyme and potential feedback on transcription is necessary for future development of drugs and to prevent the development of resistance.

## Supporting information

S1 File Teflubenzuron-treated nauplii three days post-treatment. (MOV)

S2 File Raw qPCR data from chitin synthesis inhibitor treated nauplii II larvae. (XLSX)
